# Deep Architectures Fail to Generalize: A Lightweight Alternative for Agricultural Domain Transfer in Hyperspectral Images

**DOI:** 10.3390/s26010174

**Published:** 2025-12-26

**Authors:** Praveen Pankajakshan, Aravind Padmasanan, S. Sundar

**Affiliations:** 1 UrbanKisaan, Hyderabad 500100, India; 2 Walmart Global Technology, Bengaluru 560103, India; 3 Industrial Mathematics and Scientific Computing, Indian Institute of Technology Madras, Chennai 600036, India

**Keywords:** hyperspectral, remote sensing, sustainable agriculture, spatial nearness, spectral similarity, weak supervision, few shot learning, transferability, open-set recognition

## Abstract

We present a novel framework for hyperspectral satellite image classification that explicitly balances spatial nearness with spectral similarity. The proposed method is trained on closed-set datasets, and it generalizes well to open-set agricultural scenarios that include both class distribution shifts and presence of novel and absence of known classes. This scenario is reflective of real-world agricultural conditions, where geographic regions, crop types, and seasonal dynamics vary widely and labeled data are scarce and expensive. The input data are projected onto a lower-dimensional spectral manifold, and a pixel-wise classifier generates an initial class probability saliency map. A kernel-based spectral-spatial weighting strategy fuses the spatial-spectral features. The proposed approach improves the classification accuracy by 7.22–15% over spectral-only models on benchmark datasets. Incorporating an additional unsupervised learning refinement step further improves accuracy, surpassing several recent state-of-the-art methods. Requiring only 1–10% labeled training data and at most two tuneable parameters, the framework operates with minimal computational overhead, qualifying it as a data-efficient and scalable few-shot learning solution. Recent deep architectures although exhibit high accuracy under data rich conditions, often show limited transferability under low-label, open-set agricultural conditions. We demonstrate transferability to new domains—including unseen crop classes (e.g., paddy), seasons, and regions (e.g., Piedmont, Italy)—without re-training. Rice paddy fields play a pivotal role in global food security but are also a significant contributor to greenhouse gas emissions, especially methane, and extent mapping is very critical. This work presents a novel perspective on hyperspectral classification and open-set adaptation, suited for sustainable agriculture with limited labels and low-resource domain generalization.

## 1. Introduction

Monitoring crop health, distribution, and biophysical traits through advanced hyperspectral remote sensing is a cornerstone for sustainable agricultural systems, enabling precision resource management, adaptation to climate variability, and mitigation of environmental impacts such as greenhouse gas emissions. Accurate mapping and quantification of the biophysical and biochemical traits of agricultural crops, growth seasons, land use, and human activity are crucial for sustainable agriculture and food security. These enable precise resource management, adaptation to climate variability, and mitigation of environmental impacts such as greenhouse gas emissions [1,2]. Regular temporal monitoring throughout the cropping cycle, from sowing to harvest, is essential for timely interventions. Monitoring of farm plot and land use at scale and at different time instances of a growing season, and pre-/post-harvest is very critical. Farmers, support organizations or field scientists/personnel find it difficult to audit or scout fields on a regular basis for monitoring or interventions. This has led to increased reliance on low-altitude flights, satellite-based and UAV-based remote sensing technologies [3,4]. There were many historical developments in imaging spectrometry for applications in agriculture and forestry, since the launch of Landsat 1 [5] in 1972 to the present day high-resolution imagery based on UAV sensors [1]. More recently, UAVs have been equipped with imaging spectrometers to collect high-resolution imagery for applications in agriculture and forest. Hyperspectral Imaging (HSI) [6], with its rich spectral bands, has emerged as a key enabler providing information in both the spatial, and the spectral dimensions. Using field-collected data, machine learning, and high spectral resolution, it is possible to perform crop discrimination [2], determine biotic and abiotic stresses [7,8], monitor health, nutrients [9], cropping patterns, and support the goals of sustainable development [10].

The article [11], provides the recent trends in HSI analysis, while in [12,13], the authors review recent Hyperspectral and Deep Learning methods. Deep learning-based methods [14,15,16,17,18,19] have relatively higher accuracy in comparison to other approaches. Remote sensing plays a crucial role in quantifying crop biophysical parameters and supporting sustainable agricultural practices. HSI provides detailed spectral information that enables discrimination of crop types, health conditions, and stress factors. Despite these advantages, Hyperspectral sensed images in application to agricultural applications is limited by five key challenges:

- the number of spectral components increases the data processing complexity during preparation, training and inference [6],
- high correlation between the individual bands and added redundancy [20],
- difficulty in interpreting the model [21,22] and explainability of the decision making process for trust and scaling,
- limited number of benchmark labeled datasets available after ground truth verification for the different practices followed in sustainable and regenerative agriculture, and
- limited ability of the models to generalize to new regions or new classes or new context where limited or no prior data is available for training.

Although earlier studies extensively addressed spectral redundancy, data dimensionality, and model interpretability, these methods are entirely focused on obtaining high-accuracy classification maps in a very localized or selected study region. Very few works address the last two challenges related to the limited dataset with diverse classes and generalizability of models. Notable among these previous work is [23] where the authors use a three-dimensional convolutional neural network or 3D CNN along with an OpenMax layer (with a Weibull distribution) to reject unknown classes. Unfortunately, in new geographies (test region), often there can be minimal to no overlap between the classes in the training and the test sites or classes. This can especially be the case for agricultural applications. In which case, the classifier can reject all crops of interest. In Ref. [24], the authors designed a framework to enhance the latent spectral and spatial features that can be used to classify the known classes while rejecting the unknown classes. Recent studies on trade-offs have focused mainly on pan-sharpening [25], super-resolution [26], or enhancement [27]. In Ref. [28], a Dynamic Spatial-Spectral Attention Network (DSSAN) is introduced with an adaptive attention mechanism to dynamically recalibrate features. Similarly, in Ref. [29], the authors introduce an attention mechanism as well by separating spatial and spectral as two independent branches, and incorporate modules with convolution, LSTM and attention. There is no doubt that the above and recent approaches demonstrate high accuracies in closed-set benchmark data sets [30] but the parameter space is quite large in addition to being computationally very expensive. For large scale deployment for practical applications (with full training inference class overlap), trust in the performance of these models is less due to overfit to the training data/domain. Most importantly, when operating in new regions, environment, and crops, ‘by design’ they cannot be generalized.

This study presents a novel approach and framework that addresses all of the above five challenges, with a special emphasis on domain generalization (minimal or no overlap with training classes) and low-resource adaptability. We demonstrate transferability and domain generalization for paddy extent mapping. Paddy fields play an essential role in global food security, but are also a significant contributor to greenhouse gas emissions, especially methane. Methane produced under anaerobic growing conditions typical of flooded rice cultivation accounts for approximately 30% of global anthropogenic methane emissions and approximately 9–11% of total agricultural GHG emissions. Therefore, accurate remote sensing of the extent of flooded rice is essential to estimate, monitor, and manage these emissions, thus supporting climate-smart and sustainable rice production systems. This study aims to evaluate whether a weakly supervised minimal parameter spectral–spatial classifier trained on benchmark datasets is transferable to low-label agricultural settings.

### 1.1. Problem Formulation

Let $I\in R^{M\times N\times D}$ be the HSI where *M* and *N* are the spatial dimensions and *D* is the number of spectral bands in the image. We define *K* as the number of distinct classes, with K≥2, and by $\left\{\omega_{i}\right\}$ the set of class labels that belong to the set of universal class labels Ω. I is transformed into the set of features $X\;=\;\left(x^{\left(1\right)},x^{\left(2\right)},\ldots ,x^{\left(D\right)}\right)\in R^{P\times D}$, where P=M×N is the total number of pixels. The set of training data pairs, $T\;=\;\{\left(x^{\left(1\right)},\omega^{\left(1\right)}\right),\left(x^{\left(2\right)},\omega^{\left(2\right)}\right),\ldots ,\left(x^{\left(\tau \right)},\omega^{(\tau }\right))\}$, is randomly chosen from X. For our case, we assume that the training examples τ≪P. The first objective is to build a classifier, F s.t. F:I↦ω using T, where the final class labels are $\hat{\omega }\;=\;\left({\hat{\omega }}^{\left(1\right)},{\hat{\omega }}^{\left(2\right)},\ldots ,{\hat{\omega }}^{\left(P\right)}\right)$.

### 1.2. Contributions

The key contributions of this work are as follows:

- A lightweight framework that introduces a tunable balance between spectral similarity and spatial nearness, with minimal computational overhead.
- A weighting mechanism that requires tuning of at most two dataset-specific parameters (*C*, γ) while maintaining four fixed parameters (*r*, $\sigma_{w}$, $\sigma_{s}$, β) transferable across domains, enabling operation with only 1–10% labeled data.
- Demonstration of domain generalization capabilities by training on one benchmark dataset (Indian Pines) and successfully inferring on two different datasets (Kennedy Space Center dataset and the Hyperion Botswana dataset) without retraining.
- Extension to real-world agricultural settings (e.g., ASI PRISMA imagery over Italy), showcasing transferability to unseen crops and regions with minimal supervision.
- Comparative performance exceeding or matching state-of-the-art transformer-based models (e.g., MASSFormer 97.92% with 0.5% training samples and ours 97.69% with 1% training samples in Salinas) in terms of accuracy, computational efficiency, and label efficiency.

## 2. Learning Approach

**Figure 1:**
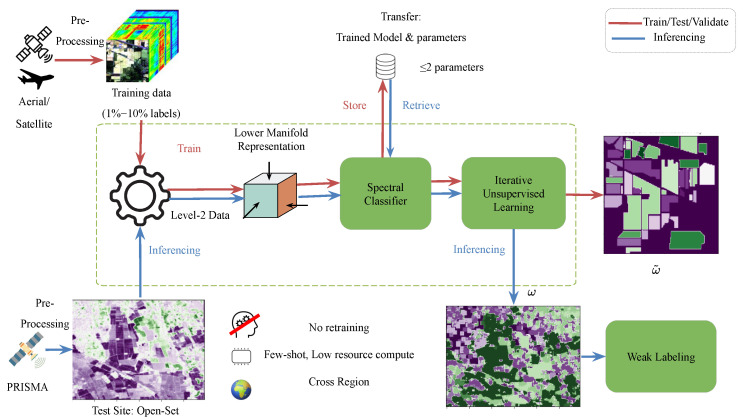
The proposed approach showing the training, testing and validation on hyperspectral images with known class labels and inferring the learned model with fixed parameters to a new test site from PRISMA. Transfer: Trained Model and parameters (no retraining required).

The proposed learning approach is shown in Figure 1 and has the following main steps:

1. Map the analysis-ready data X to a lower dimension representation z in the space S, reducing along the spectral features (either linearly or non-linearly)
2. Supervised pixel-wise classification on *K* classes on the new representation,
3. Construct the class probability map based on the pixel-wise classifier and use as initial seed
4. Iterative unsupervised learning using trade-off between spatial nearness and spectral similarity measures,
5. Ensemble the different model outputs from each iteration based on majority voting
6. Final class assignment using weak labeling.

We will discuss the details in the following subsections.

### 2.1. Class Separability

As the number of spectral bands in the hyperspectral is very high (*D*), for a spectral classifier such as a SVM, the worst-case computational complexity can be $O(D\times P^{3})$. This is computationally expensive for large features and the additional bands can add redundancy [31].

For class separability, we project the data into another manifold. After exploring many methods in our experiments and validating their performance with respect to the spectral classifier, the PCA [31,32] worked reliably with the least loss of accuracy. This is also consistent with previous work on this subject [33,34]. KPCA and other nonlinear methods gave poor results when the training samples is small or was computationally more expensive without significant contribution to the accuracy.

The data X, are orthogonally projected onto a lower-dimensional linear principal subspace S, to capture maximum data variance. The principal components are the Eigen vectors of the covariance matrix of X and the amount of “explained variance” in each component is proportional to the Eigen value. Let each hyperspectral pixel $x_{i}\in {ℜ}^{D}$ be projected onto a lower-dimensional spectral manifold $z_{i}\in {ℜ}^{d}$:

$$z_{i}\;=\;U_{d}^{⊤}x_{i},\;z_{i}\in {ℜ}^{d}$$

The explained variances are arranged in descending order of value, and the components are chosen so that the cumulative explained variance ratio is higher than a threshold $\left[0,100\right]$. We fixed the value of the threshold to be ≥90% as the cumulative explained-variance plateaued beyond.

We observed that PCA alone cannot bring about class separability. For example, in the Indian Pines dataset, the “Corn-notill”, the “Corn-mintil” and “Corn” classes seem to have very similar spectral signatures. This also applied to the classes “Soybean-notill”, “Soybean-mintill”, “Soybean-clean”. Some sustainable and regenerative agriculture farmers practice ‘zero-tillage’ or ‘minimum-tillage’ to reduce mechanical interventions to the soil so as to not disturb the physical structure or the beneficial organisms present in the soil. However, it is difficult to detect the intervention or the absence of intervention practices such as tillage using only a single time-stamp image. Interventions may have occurred before sowing, while the acquired image may have been captured much later in time during the vegetative period or at the time of maturity. When the canopy is dense, the signatures from the soil are minimal, and hence the signatures are mostly from the crops. This explains why these classes might have similar signatures when we study the spatial-spectral features.

### 2.2. Construction of Initial Classification Probability Map

The initial classification map $\tilde{\omega }\in R^{P}$ is obtained from a pixel-wise spectral classifier

(1) $$F_{\theta }^{\mathrm{SVM}}\left(z\right)=\arg\max_{k\in \{1,2,\ldots ,K\}}w_{k}^{T}\Phi \left(z\right)+b_{k}$$

with multiclass decision functions, where Φ is identity (linear) or kernel map. For each pixel, we attribute the class probability as

(2) $$p_{i}^{\mathrm{svm}}\left(k\right)≜P^{\mathrm{svm}}\left(\omega_{i}=k|z_{i}\right),\;k\in \left\{1,\ldots ,K\right\}$$

with $\sum_{k=1}^{K}p_{i}^{\mathrm{svm}}\left(k\right)=1$. The initial probability map for all pixels, $P\in R^{P\times K}$ is used as an initializer for subsequent steps. It is possible to add a penalizing term to the probability map depending on the deviation from the true labels $\omega_{i}$. There is much prior literature that compares Random Forest (RF) and Support Vector Machine (SVM) for pixel-wise labeling of the images. While the overall accuracy (OA) is comparable, SVM classifiers better spatially associate pixels with similar signatures, resulting in less noisy maps [35,36]. In addition, the number of parameters is lower than that of tree-based approaches. Throughout this text, we will use SVM as the pixel-wise classifier, but it can be replaced by RF or CNN-based classifiers with softmax probabilities. We used the SVM algorithm implemented in the ThunderSVM library [37] that has GPU support. The initialization seed (similar to the unary potentials [38,39]) is constructed from the probability map of the pixel classifier.

### 2.3. The Model

We model the joint distribution of the probabilities of pixels using the nearness with the spatial neighbors and spectral similarity. For each pixel, *i*, a neighborhood $N_{i}$ which belongs to the partition Ω of the possible neighborhood partitions of the image. This neighborhood is defined on the output obtained from the spectral classification map, and the joint probability distribution is constructed by integrating the spatial nearness and the spectral similarity between the pixels. The following are the definitions of nearness and similarities.

#### 2.3.1. Spatial Nearness Kernel

We assume that the pixels $j\in N_{i}$, ∀j≠i are spatially close. To quantify this idea, we have the spatial proximity kernel as:

(3) $$S_{i}\;=\;\exp\left(\frac{-{∥j-i∥}_{2}^{2}}{2\sigma_{s}^{2}}\right)$$

where, ${∥\cdot ∥}_{2}^{2}$ is the distance $l_{2}$-norm between the reference pixel *i* and the neighborhood set of pixels j. The parameter of the Gaussian kernel $\sigma_{s}$ controls the local spatial connectedness of the pixels. A lower (higher) value of $\sigma_{s}$ means that the pixels that are closer are only (farther are also) considered spatially similar to the central pixel. This also ensures label compatibility between neighboring pixels.

#### 2.3.2. Spectral Similarity Kernel

We can assume that the spectrum of the reference and neighborhood pixels is similar if they are of the same class. The spectral similarity is defined as follows:

(4) $$W_{i}\;=\;\exp\left(-\frac{{∥f_{i}-f_{j}∥}_{2}^{2}}{2\sigma_{w}^{2}}\right)$$

where, $f_{i}$ is the spectral feature vector of the reference pixel, $f_{j}$ is the spectral feature of the neighborhood pixels in j, and $\sigma_{w}$ is the parameter that controls the degree of similarity. Due to normalization, the parameters of the kernels $\sigma_{s}$ and $\sigma_{w}$ are independent of both the spatial and spectral resolutions, respectively.

#### 2.3.3. Boundary Condition Handling

For pixels, *i*, at the image boundaries where the full (2r+1)×(2r+1) neighborhood extends beyond the image extent, we employ mirror padding:

1. Symmetric Padding: The input image $I\in R^{M\times N\times D}$ is padded to $\tilde{I}\in R^{(M+2r)\times (N+2r)\times D}$ by reflecting the pixel values at the borders. This ensures that every pixel has a complete ${\left(2r+1\right)}^{2}$ neighborhood and is better than truncation.
2. Small Memory Overhead: For r=4, the padding increases only fractionally the memory footprint and is negligible.

#### 2.3.4. Attention as Generalized Weighting

In recent transformer architectures, self-attention computes pairwise relationships (attention weights) between spatial tokens/pixels, dynamically weighting their influence based on feature similarity. The kernels described in the previous paragraphs $S_{i}$ (spatial proximity) and $W_{i}$ (spectral similarity) play similar roles as attention masks that highlight the relative importance of neighboring pixels based on their spatial proximity and spectral relatedness. Many of the vision transformers (e.g., Swin transformers) incorporate a variant of localized self-attention to the spatial windows, and they locally resemble spatial kernels while the spectral similarity kernel promotes semantic consistency.

#### 2.3.5. Unsupervised Learning

We construct the conditional probability distribution of the final classification map (ω), given the initial classification map ($\tilde{\omega }$) and the unsupervised learning parametrized by $\sigma_{s}$ and $\sigma_{w}$. i.e., $P(\omega |\tilde{\omega };\sigma_{s},\sigma_{w})$. Since the adjacency of pixels in the image is a local phenomenon, it might not be necessary to use the entire initial classification map to obtain the final classification label for a pixel. For the reference pixel *i*, we compute the conditional probability in the neighborhood N(i). This approach reduces computational cost by not using the predictions from pixels far from the reference pixel [40]. Using the definition of conditional probability,

(5) $$\begin{array}{c} P\left(\omega_{i}|{\tilde{\omega }}_{N(i)};\sigma_{s},\sigma_{w}\right)\;=\;\frac{P(\omega_{i},{\tilde{\omega }}_{N(i)};\sigma_{s},\sigma_{w})}{P({\tilde{\omega }}_{N(i)})} \end{array}$$

The numerator term in the above equation is the joint distribution of the initial and final classification maps. Since the denominator term is constant over the neighborhood, the numerator is the only component which decides the conditional probability. In Ref. [39], the authors use a mean-field approximation to compute a distribution $Q(\omega_{i})$ rather than to compute the exact distribution of Equation (5).

To make the computation of the joint probability tractable, we rather assume conditional independence between the neighborhood (given the reference pixel), and we factorize the distribution based on this assumption. Let us suppose that *j* and *l* are the neighborhood pixel locations in $N_{i}$, the conditional independence implies that $\omega_{j}\;⊥\;\omega_{l}|\omega_{i}$. So, using this condition, the joint probability distribution can be factorized as the following:

(6) $$P\left(\omega_{i},{\tilde{\omega }}_{N(i)};\sigma_{s},\sigma_{w}\right)\;=\;\frac{1}{Z}\prod_{j\in N(i)}\psi (\omega_{i},\omega_{j})$$

where $\psi (\omega_{i},\omega_{j})$ is the potential function between the reference pixel *i* and the neighborhood pixel $j\in N_{i}$, and *Z* is the partition function given by,

(7) $$Z\;=\;\sum_{i\;=\;1}^{P}\prod_{j\in N_{i}}\psi (\omega_{i},\omega_{j})$$

The potential function in Equation (6) can be any positive non-zero functions. Since we are restricted to strictly positive functions, it is convenient to express the potential functions as an exponential:

(8) ψ(X)=exp(−E(X))

We assume that E(X) is the energy function which is influenced by the unary potentials of the spectral classifier and the neighborhood interactions.

The joint distribution is defined as the product of potentials, and so the total energy is obtained by adding the potentials of the interactions in the spatial and the spectral directions. The potential is defined as (below all the multiplications are element-wise):

(9) $$\begin{array}{c} \phi_{i}\left(X\right)\;=\;-\left(1-\beta \right)\left(W_{i}+S_{i}\right)P_{j}-\beta I\left(\omega_{i},\omega_{j}\right) \end{array}$$

where the indicator operator $I(\omega_{i},\omega_{j})$ is given by,

$$I\left(\omega_{i},\omega_{j}\right)\;=\;\left\{\begin{array}{cc} 1 & \mathrm{if}\;\omega_{j}\;=\;\omega_{i}\\ -1 & \mathrm{otherwise} \end{array}\right.$$

β≤1 is the parameter of the energy function and $P_{j}$ is the class probability at neighbor pixel *j*. By combining the spatial and the spectral components together, we can rewrite Equation (9) as:

(10) $$\begin{array}{c} \phi_{i}\left(X\right)\;=\;-\left(1-\beta \right)W_{i}P_{j}+\beta \left(S_{i}P_{j}-I\left(\omega_{i},\omega_{j}\right)\right) \end{array}$$

By replacing β/(1−β) by $\beta^{\prime }$, this could be simplified as:

(11) $$\phi_{i}\left(X\right)\;=\;\underset{\mathrm{spectral}\;\mathrm{similarity}\;\mathrm{term}}{\underset{︸}{W_{i}⊙P_{j}}}-\beta^{\prime }\cdot \underset{\mathrm{spatial}\;\mathrm{nearness}\;\mathrm{term}}{\underset{︸}{\left[S_{i}⊙P_{j}-I\left(\omega_{i},\omega_{j}\right)\right]}}$$

where: · denotes element-wise multiplication, $W_{i}$ spectral similarity weights (high for spectrally similar neighbors), $S_{i}$ spatial proximity weights (high for nearby neighbors), $P_{j}$ class probabilities from initial SVM, $I(\omega_{i},\omega_{j})$ is an indicator function (1 if same class, −1 otherwise), $\beta^{\prime }$: trade-off parameter controlling spatial closeness vs. spectral similarity. The above is familiarly similar in form to the way that the unary and pairwise potentials are built for the Gibbs’ energy [38]. The difference is that in this case, the output from the spectral classifier interacts with the nearness and similarity kernels non-linearly and is not a spatial-only regularizer. As described earlier, the total energy is the sum of the potentials as

(12) $$E_{i}\left(X\right)=\sum_{j\in N(i)}\phi_{i}\left(X\right)$$

From Equation (11), the parameter $\beta^{\prime }$ gives a trade-off between the spatial nearness metric and the spectral similarity metric. For simplicity herein we refer to $\beta^{\prime }$ as β. This parameter is linear in the cost function and can be estimated by minimizing the cost function in β. Since the initial classification has good accuracy, we weigh the probability map of the reference pixel with spatial nearness and spectral similarity to reward or penalize the correct and incorrect classification of the initial spectral classifier.

To make a prediction on the reference pixels, we now fix the initial probability map based on the spectral classifier, which implicitly defines the conditional distribution $P(\omega |\tilde{\omega };\sigma_{s},\sigma_{w},\beta )$. First, we fix the final classification map to the initial classification map. Then, we take one reference node *i* at a time and evaluate the total energy for all possible states, keeping all other variables fixed, and set the reference node *i* to whichever state has the lowest energy. Since the entire inner-loop computation is performed in a small neighborhood, the overall optimization is efficient. After finding the state for a node, we will move to another node and the above steps are repeated. We have a sequence of updates in which every site is visited at least once, and the variables remain unchanged during this time. This inner site-iteration is repeated until the assignment of the class labels is complete for all the sites. The entire methodology is repeated as an outer loop iteration (*n*) until the total assignment probability of the class labels stabilizes i.e.,

(13) $$\sum_{i}∥P{\left(\omega |\tilde{\omega }\right)}_{i,n+1}-P{\left(\omega |\tilde{\omega }\right)}_{i,n}∥\leq \epsilon$$

where $P{\left(\omega \right)}_{i,n}$ refers to the probabilities of the class at site *i*$P{\left(\omega |\tilde{\omega };\sigma_{s},\sigma_{w},\beta \right)}_{i,n+1}$ at the $n^{th}$ iteration and ϵ is a convergence threshold that can be very small (say $\;10^{-4}$). This iterative approach has been explored in other prior work as in the ICM [35]. The inner and outer loop iterations ensure that the local and global connectivity of the pixels are maintained. A section of the Python 3.12.12 notebook and the benchmark dataset is made available for reproducibility as a Github (https://github.com/praveenpankaj/mdpi_sensors_hsi/, accessed on 16 October 2025) and newer versions will be uploaded to IEEE CodeOcean [41].

### 2.4. Ensemble Voting

An ensemble voting algorithm helps in generalizing the model well and avoids overfitting to the training samples [19]. This can decrease the overall accuracies, but ensures the reliability of the classification results. So, the following optional steps are proposed in addition to the above methodology.

- Step 1: The features are divided into 4 mutually exclusive and collectively exhaustive subsets using random sampling.
- Step 2: The proposed model is built on all the subsets individually and the classification map is obtained for every single model.
- Step 3: The classification maps from Step 2 are stacked and the final classification map is obtained by voting for the majority class.

## 3. Evaluation

### 3.1. Benchmark Data for Training and Testing, and Open-Set Data for Inference

**Figure 2:**
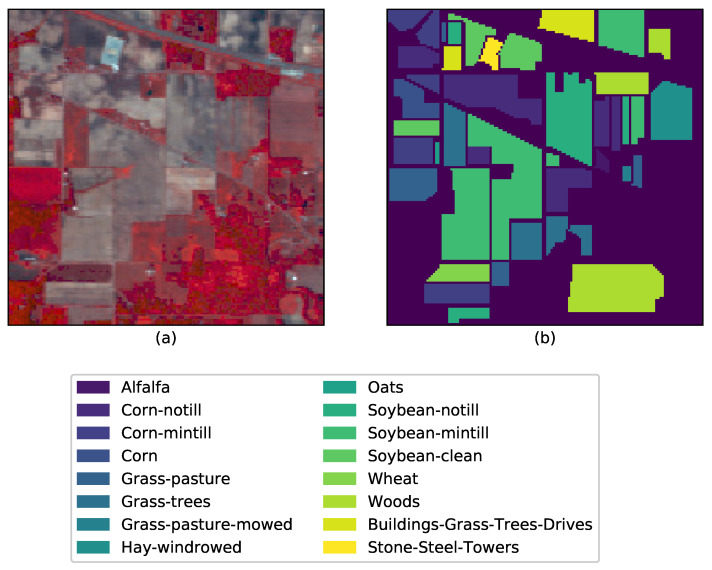
AVIRIS Indian Pines data set and the corresponding ground truth data: (**a**) Band 3 (central wavelength 419.62, FWHM 9.85, (**b**) Ground truth with class labels.

We use data from three open-source hyperspectral images, which are the Indian Pines Image [42], the Salinas Image [42], and the University of Pavia Image. These are from different sensor resolutions, sizes, and classes. The Indian Pines’ scene was captured by NASA’s AVIRIS sensor over the test site in North Western Indian, USA [43]. The image has a size 145×145 with a spatial resolution of 20 m and a total of 224 spectral reflectance bands present within the wavelength range 0.4 μm to 2.5 μm. The scene mainly contains agricultural land, forests, grasslands, and some urban classes. The ground truth is mapped into sixteen classes that are not all mutually exclusive. It is a conventional practice to reduce the number of spectral bands to 200 by removing the bands covering the regions of water absorption e.g., the bands [104−108],[150−163],200. Figure 2 shows a randomly selected band (band 3) and the ground truth with class labels.

**Figure 3:**
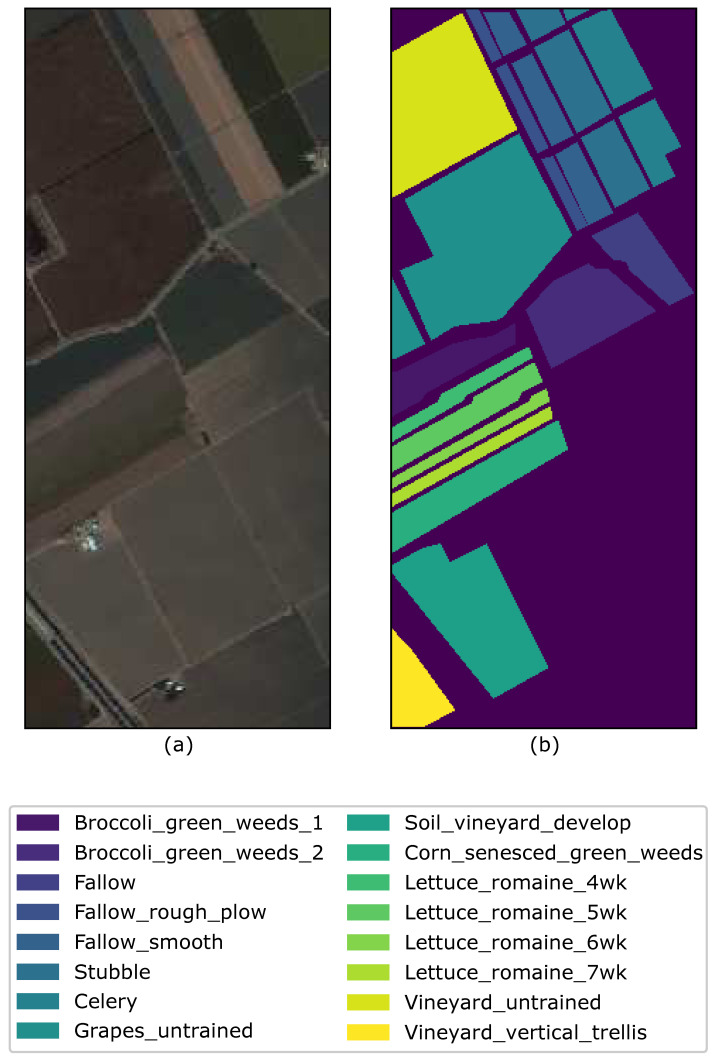
(**a**) Band 101, (**b**) Ground truth of the Salinas image with the respective class labels.

The Salinas image was captured by NASA’s AVIRIS sensor over the Salinas Valley in California. The image has a size 145×145 with a spatial resolution of 3.7 m and has 224 spectral reflectance bands. Similarly to the Indian Pines, there are twenty bands that cover the water absorption bands with band numbers [108−112],[154−167],224. The Salinas ground truth is designated to sixteen classes that are not all mutually exclusive. The Salinas scene contains vegetables, bare soils, and vineyard fields. Figure 3 shows a randomly chosen band 101 with ground-truth.

**Figure 4:**
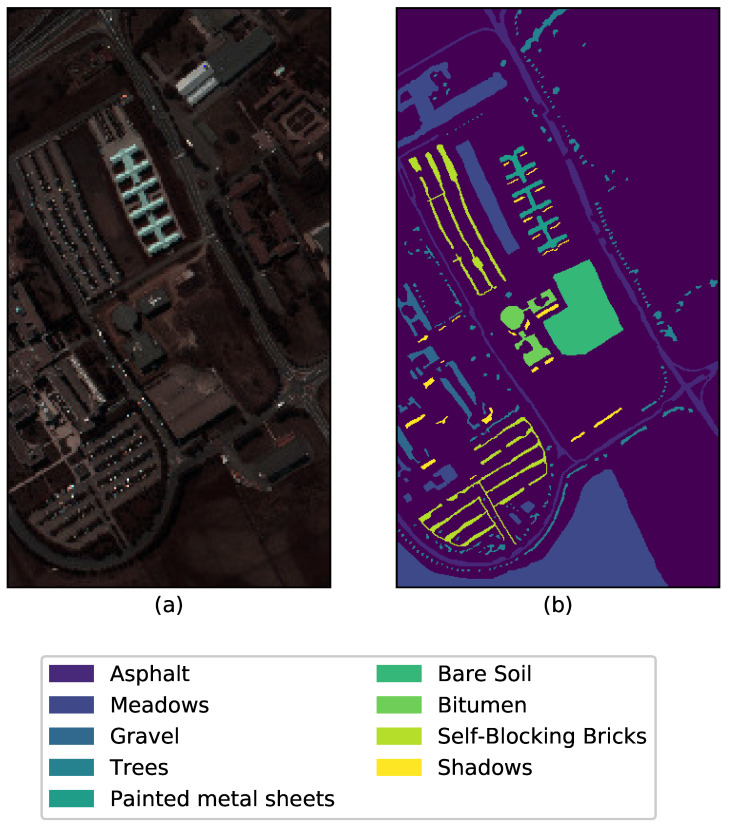
(**a**) Band 41 and (**b**) Ground truth of the Pavia University data with the respective class labels.

The Pavia University scene was acquired as one of the experimental analyses using the ROSIS-03 sensor (or Reflective Optics System Imaging. Spectrometer) during a flight campaign over Pavia in Northern Italy. The ROSIS was jointly developed by Dornier Satellite Systems (DSS, former MBB), GKSS Research Centre (Institute of Hydrophysics) and German Aerospace Center (DLR, Institute of Optoelectronics). The detector chip is a 2-dimensional CCD array. The image has 115 spectral bands of size 610×340 and with a spatial resolution of 1.3 m per pixel. It is the general norm to keep only 103 channels from the original 115 by discarding the 12 most noisy channel from the raw data. The ground truth contains nine classes as shown in Figure 4 with a randomly chosen band 41.

The test samples are chosen from the same 3 set of images, and hence this is a closed-set with the class representations in the test being fully represented in the training set.

The open-set data are chosen from a study region in Italy, as described in Section 3.6. The PRISMA sensors acquire VNIR and SWIR products, with a 30 m spatial-resolution, and a panchromatic camera with a 5 m spatial resolution. The hyperspectral camera works in the range of 0.4–2.5 μm with 66 and 173 channels in the VNIR and SWIR, respectively. Due to their low SNR, SWIR bands B158 (2392 nm) and B173 (2495 nm) were not considered. We used the L2D HCO product and the SWIR images with the number of bands being 171 and a size of 214×297.

### 3.2. Performance Metrics

The performance of the proposed model is evaluated using three quality indices, which are the overall accuracy (OA), average accuracy (AA), and the Kappa coefficient. The OA is the percentage of correctly classified pixels, the AA is the mean of the percentage of correctly classified pixels for each class, and the Kappa coefficient gives the percentage of correctly classified pixels corrected by the number of agreements that would be expected purely by chance.

The advantages of the framework are also computational efficiency. In Ref. [29], the authors have made a comparison of the different state-of-the-art algorithms and have highlighted that the SVM with the RBF kernel has the least computational time during training. The computational order for our approach is determined by the pixel classifier viz. $O(N^{2})$, while for CNN it will be O(P×L×M) for *P* pixels, *L* layers and *M* operations in each layer.

### 3.3. Analysis of Parameters

The proposed methodology is a mixture of supervised and unsupervised classification because the first stage of the modeling is a supervised spectral classification, and the second stage is an unsupervised approach that uses the spatial nearness and spectral similarity of pixels in the image. The basic objective of the proposed model is to designate each pixel in the image to a class by making use of the least amount of labeled data. So, the entire pixels in the image are split into two sets, viz. training data and testing data s.t. the training data have samples from each class. The labels of the first set are visible to the model and are used to determine the optimum value of parameters of the classifier, and the testing data is left untouched. The parameters of both classifiers are determined independently and the methodology is as follows.

**Figure 5:**
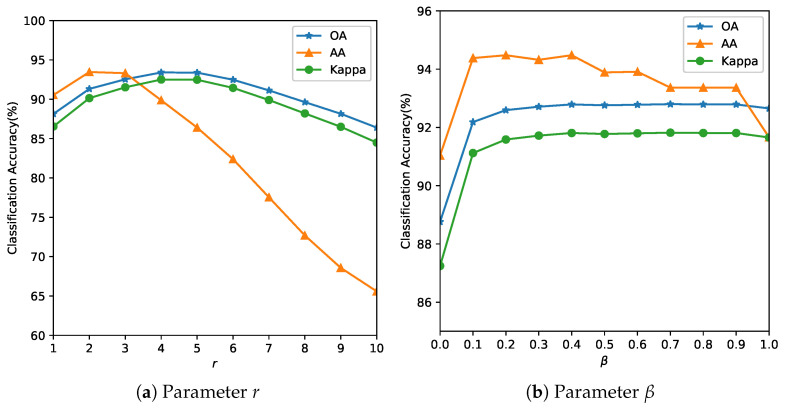
Analysis of the influence of parameters *r* and β for the Indian Pines Image. The optimal r=4 balances spatial consistency with edge preservation. Smaller values (r<3) produce noisy classifications; larger values (r>6) over-smooth field boundaries.

**Figure 6:**
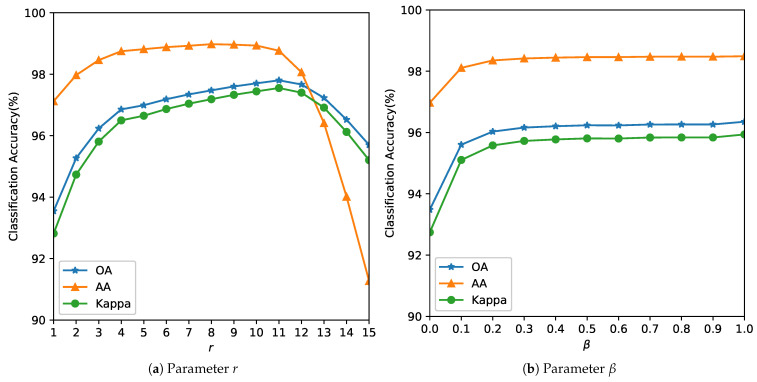
Analysis of the influence of parameters *r* and β for the Salinas Image.

**Figure 7:**
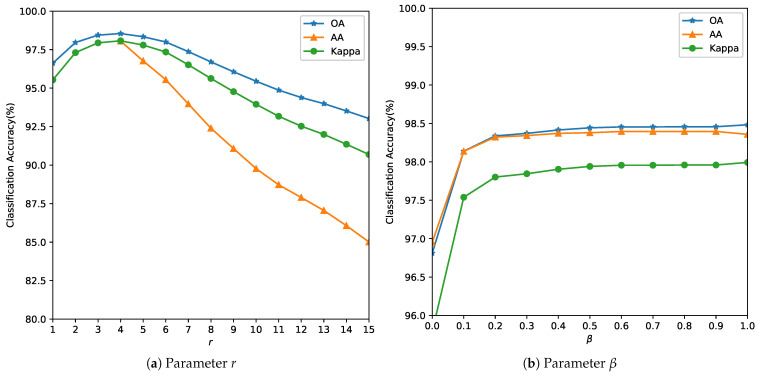
Analysis of the influence of parameters *r* and β for the University of Pavia Image.

**Figure 8:**
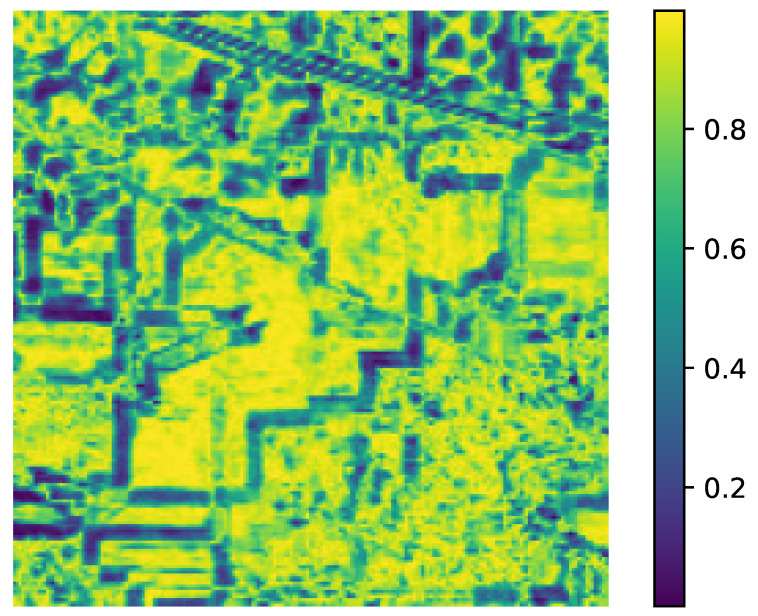
Spectral Similarity in the AVIRIS Indian Pines data for the parameter value ($\sigma_{w}\;=\;1.3$). High values are regions with high spectral similarities and vice versa.

1. Parameter Selection for PCA: We fixed the PCA parameters, viz. the explained ratio, to 0.92 for all images, and the optimal number of principal components can be automatically calculated from that threshold. The cumulative explained variance ratio curves reached saturation after the first 30 main principal components. However, during cross-validation (CV), we found that the accuracies of the classifier do not improve after the first 18 of the principal components. For the training data partition, we deliberately used a smaller training fraction than conventional literature splits in order to simulate real-world data-scarce agricultural conditions (see Table 1).
2. Spectral classifier parameter tuning: We used SVM [36] with the RBF kernel as a spectral classifier due to its relatively higher accuracy compared to other classifiers. We observed that spatial adjacency is maintained better by SVM’s pixel-level class label assignment than by using a tree-based classifier. When training, we randomly choose a few pixels from each class label. For training data partition, we deliberately used a smaller training fraction than conventional literature splits in order to simulate real-world data-scarce agricultural conditions:
  This choice is based on our operational experience:
  - ground truth collection requires expensive field campaigns and multiple visits during a growing season,
  - the minor crops that are grown in a region during main season could be minimum,
  - with changing climatic conditions, for the same region, a different crop might be chosen the next season and re-labeling is required.
  Our objective is to demonstrate that the proposed method maintains competitive accuracy even under extreme label scarcity (1%), whereas deep models typically require much higher data for stable training.
  As the number of parameters of the SVM (*C* and γ) is small, these are estimated using an exhaustive grid search algorithm [44,45] within a specified coarse bound using a 5-fold CV. Some authors have estimated that their algorithm gave the best OA when 10% of data was used for training for the Indian Pines, 4% for the Salinas and 6% for the Pavia respectively [46]. In real-world scenarios, ground truth is sparse and expensive. We used smaller training fractions than conventional splits for these benchmark datasets to highlight low-label scenarios. So, we reduce efforts in manual ground truth data collection and annotation, our objective towards model building was to use as few training samples as possible or low-resource conditions. Finer grid search for the parameter is computationally feasible and can be parallelized [44] but at the risk of overfitting to the data.
3. Parameter tuning: The parameter *r* determines the size of the neighborhood around the reference pixel, the parameters $\sigma_{s}$ and $\sigma_{w}$ control the degree of spatial nearness and spectral similarity Gaussian kernel, respectively. The parameter β, with 0≤β≤1, specifies the relative importance between the two terms in Equation (10). It was observed that the parameter *r* more influences the model and this is estimated first, while $\sigma_{w}$ and $\sigma_{s}$ are bounded by *r*. This is also expected behavior, as the value of the parameter *r* is linked to spatial resolution. In missions with lower resolution acquisitions, to avoid smoothing, a lower value of *r* is required. The spatial resolution and the choice of the neighborhood are also closely linked to the type of farm holding size. It is likely that for smaller landholding sizes (<1.58 acres or 6400 m^2^), the performance of the method could be affected. The performance of the model is evaluated with respect to the three quality indexes mentioned in Section 3.2 and the results are summarized in Figure 5, Figure 6 and Figure 7. The parameter *r* determines the size of the neighborhood around the reference pixel, the parameters $\sigma_{s}$ and $\sigma_{w}$ control the degree of spatial nearness and spectral similarity (see Figure 8) Gaussian kernel, respectively.

Table 2 summarizes the optimal parameters required by the algorithm for the analysis in Figure 5, Figure 6 and Figure 7. We found that by fixing the set of parameters to γ=7, r=4, $\sigma_{w}=1.3$, $\sigma_{s}=1.3$ and β=0.4, for all images, there was a slight loss in the OA but we benefit from not having to fine-tune these parameters in the future. This was also observed in [39] with the smoothness kernel parameter and the weights that did not influence the accuracy of a given dataset. So, the only parameters to estimate are the parameters of the SVM *C* and to improve accuracies the γ [47] parameter. We observed that the AA of the algorithm is especially sensitive to *C* and the degree of dependency is smaller on the other parameters, so we recommend estimating the value of *C* for each dataset.

### 3.4. Classification Results and Model Transferability

The performance of the proposed algorithm is shown, for the three data sets, in Table 3, Table 4 and Table 5 and compared with the other methods. The comparison is made with some closely related hyperspectral classification methods such as Edge Preserving Filter (EPF) [48], Image Fusion and Recursive Filtering (IFRF) [46], Random Multi-Graphs (RMG) [49]. EPF generates pixel-wise classification maps and handles these maps by edge-preserving filtering. Then, the class of each pixel is selected on the basis of the maximum probability. The IFRF combines the spatial and spectral information through image fusion and recursive filtering. IFRF does not directly extract the features of the patches and uses two parameters $\delta_{s}$ and $\delta_{r}$ to extract the spatial features. RMG is a semi-supervised ensemble learning method based on Random Multi-Graphs.

**Figure 9:**
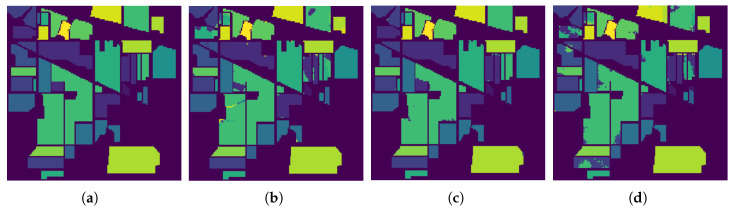
Classification results by the different methods on the AVIRIS Indian Pines data. (**a**) Ground Truth map; (**b**) EPF; (**c**) IFRF; (**d**) Proposed Approach.

**Figure 10:**
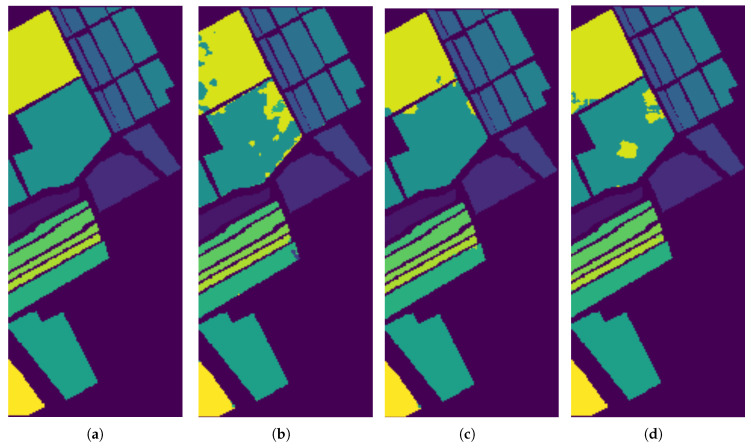
Classification results by the different methods on the Salinas image. (**a**) Ground Truth map; (**b**) EPF; (**c**) IFRF; (**d**) Proposed Approach (single run).

**Figure 11:**
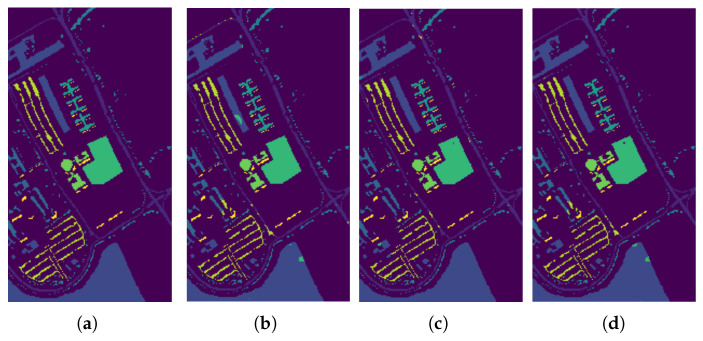
Classification results by the different methods on the ROSIS−03 Pavia University image. (**a**) Ground Truth map; (**b**) EPF; (**c**) IFRF; (**d**) Proposed Approach.

In Figure 9, Figure 10 and Figure 11, we present the classification results of the EPF, IFRF, and our proposed approach for a single run. Experimental results on this dataset show that the spatial consistency is roughly preserved by all these methods and they all significantly outperform the unary spectral classifier.

IFRF and RMG have comparable results and they slightly outperform EPF. In some of the previous work above, noisy channels were removed prior to spectral classification. In this work, the only preprocessing step was the removal of the background pixels that did not have any classification label assigned to them (mixture of classes), and the other step was to normalize the intensities (Min-Max). For the Salinas dataset, when the final classification map is fed back as weak labels, and input as a replacement for the spectral classification map, our accuracy increased to 99.42% which is the highest compared to EPF, IFRF or RMG. We observed the same phenomenon in the Indian Pines, where the accuracy rose to about 97% (from 92.78%) with just one additional run. The accuracy increased to 99.58% for the Indian Pines dataset when the number of training samples was increased. However, the greatest confusion was between the crop classes “Soybean-mintill” and “Corn-notill”. The algorithm is likely to have recognized the corn residue from the previous season as the current season planted soybean due to the minimum tilling agricultural practice. For Indian Pines, Salinas, and Pavia datasets, several new results have set accuracy benchmarks using CNNs and Transformer architectures, respectively. For example, in SSRN [16], the authors used about 25% of the samples for training for Indian Pines and about 11% for Pavia University datasets and reached maximum accuracies of more than 97% and more than 99% for Indian Pines and Pavia University, respectively, by using Batch Normalization and Dropouts. The training time for Indian Pines is longer at 106 minutes and inference is 17.2 s on a MSI GT72S laptop (Micro Star International Co Ltd., Taiwan, China) with Intel Core i7 processor up to 64 GB memory and the GeForce GTX 980M GPU 8/4GB GDDR5. SpectralFormer [50] has demonstrated accuracy > 99% with a combined training and inference time of about 20 s on a server equipped with an Intel Xeon Silver 4210 2.20-GHz CPU and an NVIDIA GeForce RTX 2080Ti GPU with 11 GB of memory. Our accuracy results are comparable and sometimes better than some of these models. The parameter complexity is very high in some of these recent models, low model interpretability, the transferability and open-set classification is not tested, and requires GPUs for deployment. In addition, in our methodology, the number of parameters to estimate are 2 (*C* and γ). Our algorithm does not require high-performance computations or GPUs, and training and inference take less than 21 s for the Indian Pines Dataset, which could be optimized further. The server CPU used is Intel(R) Xeon(R) CPU 2.20 GHz and 12.7 GB Memory. Hybrid CNN models, 2D and 3D work very well, but seem to be sensitive to the amount of training data and window size. For example, for the Indian Pines dataset, when the training data is less than 10%, for a window size of 25, hybrid approach produce an AA of 97.55%. When the window size is decreased to 9, the AA drops to 86.3%.

The model is designed to enable the transfer of all parameters except *C*, which requires dataset-specific tuning. This significantly reduces the dependency on the training data and decreases the training time since only one parameter out of six needs to be optimized for new datasets. To understand the transferability of the model, the parameters φ, were fixed at γ=7, r=4, $\sigma_{s}=1.3$, $\sigma_{w}\;=\;1.3$, and β=0.4 as described in Table 2, and the tests were carried out on the AVIRIS Kennedy Space Center dataset and the Hyperion Botswana dataset. For the Salinas-A dataset, the OA and AA were 100% at C=43. For the Botswana dataset, the true value of *C* is around the value of 32, and with this value, the AA and κ for the spectral classifier were 90.1% and 93.9%, and increased with the spectral-spatial classifier to 97.4% and 98.3% respectively. However, for the KSC image, the true value of *C* was ≫ 32, and the OA and κ values increased from 85.2% and 94.6% to 97.1% and 99.2% respectively. By setting the parameter β to a low value, the spectral classifier’s decisions alone can be included, and the performance is closer to the spectral classifier.

### 3.5. Ablation Studies

To evaluate the contribution of each component in the proposed framework, we performed ablation studies by comparing three variants:

- a pixel-wise classifier based on SVM,
- the SVM classifier followed by CRF-based regularization, and
- the full pipeline integrating weak supervision and iterative unsupervised learning with refinement.

The goal of the ablation experiments is to disentangle the importance of the components in weakly labeled guided iterative learning.

From the ablation studies, it was found that the baseline SVM classifier achieves good separability for certain dominant classes, but suffers from noise and fragmented predictions. Incorporating CRF-based regularization (SVM+CRF) provides spatial consistency and improved accuracies in several classes (e.g., Classes 2, 5, 10, 11, 12), although performance degrades in very small or spectrally ambiguous classes (e.g., Classes 1, 7, 9) leading to an OA of 91%. The complete model also takes advantage of weak labeling and iterative unsupervised learning, producing the most balanced performance across classes and significantly higher overall accuracy 93%.

These results confirmed the following.

- Purely pixel-wise classification is insufficient for open-set agricultural scenarios due to high within-class variability and due to minimal/no class overlap with training data.
- Adding spatial regularization using CRF improves the homogeneity of the predictions, but can also decrease the performance if the initial labeling is wrong. Regularization cannot handle unknown classes.
- The full model makes use of both spatial proximity and spectral similarity. So, even if the initial mapping is mislabeled, iteratively it is refined. It is also effective in handling unseen or under-represented classes, demonstrating robustness to domain transfer with limited training labels.

Overall, ablation experiments confirmed that each stage contributes incrementally, with the final system offering a practical trade-off between accuracy and generalization.

### 3.6. Testing on New Region and on Open Set Data

In Computer Vision, Open-Set is the problem of handling images or inputs having ‘unknown’ classes. Some of the classes may be absent in the training dataset, but are present in the test dataset (novel) or vice versa. Traditionally, classifiers assume inherently that the classes present in the training are also present in the test set. In the case of agricultural applications, this is not always the case. Often, the crops that have to be identified are usually novel and specific to the region of interest i.e., $\omega_{train}\cap \omega_{test}=Ø$. Depending on the agroecological zones (see https://gaez.fao.org/, accessed on 16 October 2025), there can be multiple crops that grow in a particular region during the major and minor seasons. Even if there are the same set of crops, we often find that the genetic variety may be different at the test site than at the training site, which might result in the same crop having a different spectral signature even at the same phenological stages. In addition, the type of sensor may not be the same as the availability of the same satellite modality, and coverage may not be possible at all study locations. Some recent interest has been in the development of methods to overcome the limitations of supervised methodologies in a new region or crop by fine-tuning selected collected ground data points [51]. However, it is difficult to collect sample data points across multiple seasons and multiple crops or land use patterns. There are some recent attempts at unsupervised domain adaptation methodologies [52] and transfer learning techniques, but there are no known frameworks for hyperspectral analysis for agricultural applications. All these prior limitations are motivation factors to create the framework explained in Section 2. To assess the generalizability and transferability of our method, we trained on the benchmark datasets and evaluated it at a new location, which includes multiple types of crops not seen during training i.e., $\omega_{train}\cap \omega_{study}=Ø$.

The chosen study area is in the Barengo commune, in the province of Novara, in the Italian Piedmont region, located about 80 kilometers from the city of Turin. The cumulative annual rainfall in this region is about 600 mm and the temperatures range between 5–25 °C.

**Figure 12:**
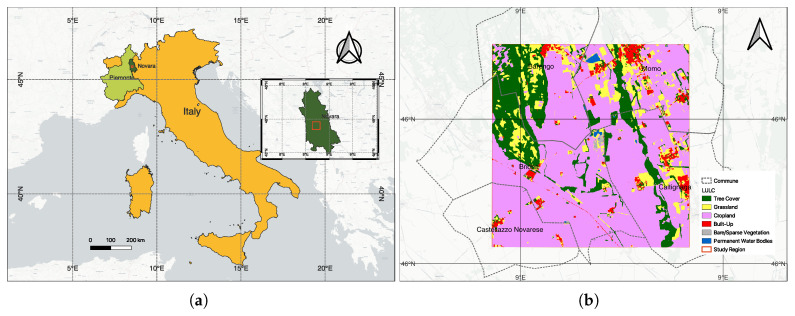
Study area. (**a**) Location map showing Piedmont region in light green and Novara province (80 km from Turin) in dark green, Italy (in yellow). Inset: Novara province (in dark green) with the study region marked in red. (**b**) ESA WorldCover 2020 land use [53] showing cropland (pink), forest (green), grassland (yellow), urban (red), water (blue), bare soil (gray). Coordinate system: EPSG:4326.

Currently, there are no field survey data for the April 2020 acquisition over Piedmont. We emphasize that the PRISMA experiment demonstrates feasibility rather than benchmarking accuracy. We therefore rely on the multi-sensor data to validate the results. We know that the study area comprises a mixture of landcover classes [53] including croplands (mainly paddy), trees, grasslands, urban/built-up areas, bare soil and a permanent water body (see Figure 12b). This is very different from the Indian Pines dataset where the crops are primarily Alfalfa, Corn, Oats, Wheat, and Soybean (Figure 2). The only common land use classes in these two datasets are grasslands/pastures and trees. We chose the Piedmont region in Italy as it is responsible for the production of almost 48% of Italy’s annual total rice production and due to the availability of hyperspectral data from the PRISMA (PRecursore IperSpettrale della Missione Applicativa) [54] (a Hyperspectral/Panchromatic instrument from the ASI-Italian Space Agency). Rice is an important crop for food security and also for sustainability. It is important to map paddy growing regions and monitor them to ensure sustainable production considering the fact that paddy is heavily dependent on water during the different stages and it also produces GHG emissions from Methanogenesis. The mapping of the extent of the rice plot, the quantification of water usage, and emissions can help plan the mitigation steps around the emissions [55]. The PRISMA image was acquired on 25 April 2020, and it is the peak period of transplantation. After land preparation and flooding, paddy transplantation begins in early April and continues until the end of May, while all harvesting is complete by mid-October. Due to climate change, there are recent variations in the beginning and end of the seasons.

**Figure 13:**
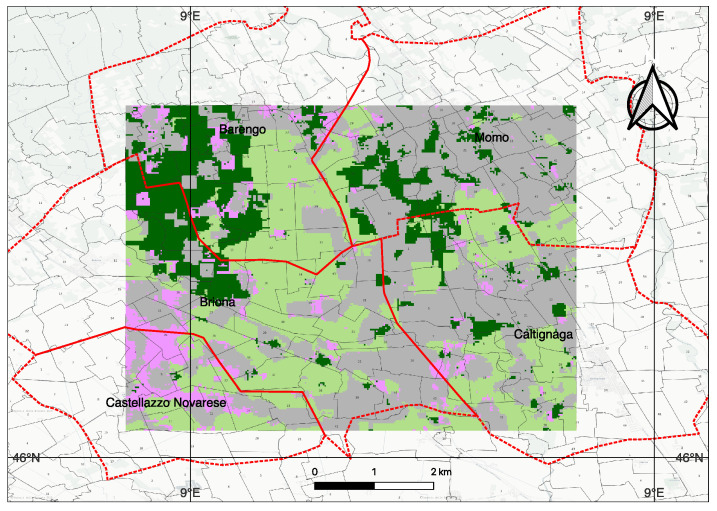
Output generated by our methodology trained on the Indian Pines dataset and inferred on the PRISMA Hyperspectral dataset at 30m spatial resolution. The groups are relabeled to the known classes of tree (dark green), crops (pink), flooded paddy (light green), barren or sparse vegetation or built-up area (gray). The methodology of labeling is given in the text. Red outline is the 5 communes (Barengo, Briona, Castellazzo Novarese, Momo, Caltignaga), and the cadastral maps. Weak labels were assigned using multi-source protocol (Section 3.6) ESA WorldCover, Sentinel-1 rice mask, Sentinel-2 NDVI, and Street View validation.

In Figure 13, we show the results of the model trained on the Indian Pines datasets but inferred on the PRISMA dataset that was specified above. The groups were ‘weakly labeled’ by subject matter experts (human in the loop), based on information from the Corine land cover [56], the ESA LULC Map [53] and ground validation.

**Figure 14:**
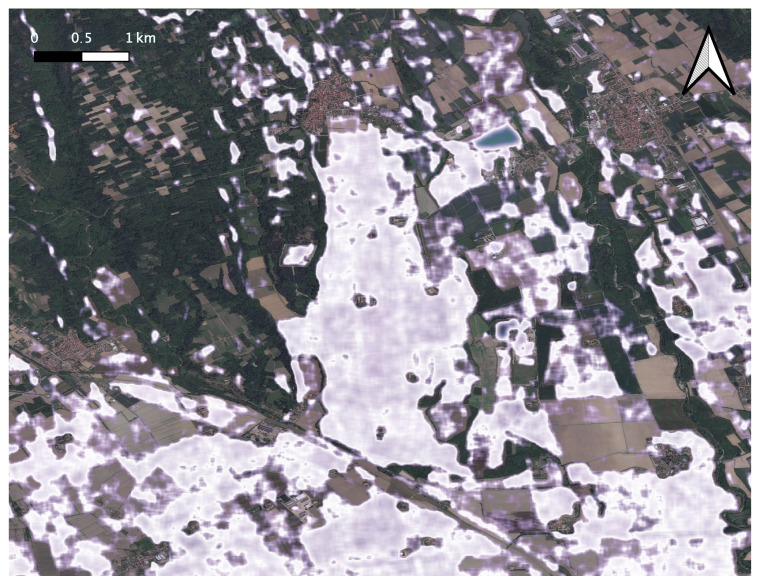
Paddy growing regions (shown in white over the basemap) identified in the summer season of 2020 from the radar backscattering coefficients of Sentinel-1 obtained from April 2020 onward for the study area shown in Figure 12. There is a very good overlap with the output generated by our methodology, shown in Figure 13.

Transplanted rice cultivation requires standing water, which serves as a spectral signature to identify the regions that grow paddy [57]. In Figure 14, we show the output rice growing region identified using the Sentinel-1 VH band as a feature layered on top of the estimated crop-land extents (pink color in Figure 12).

**Figure 15:**
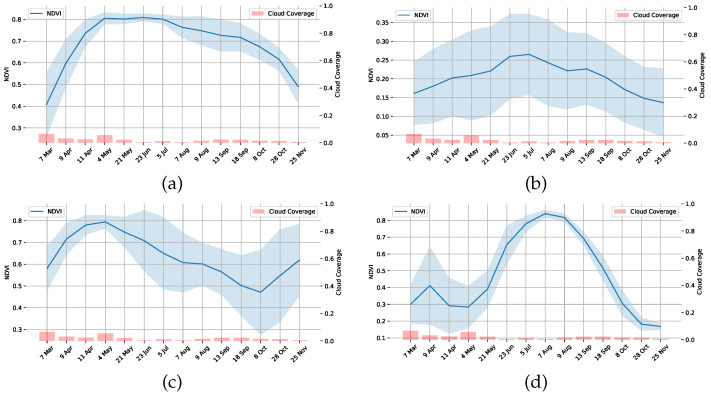
Copernicus Sentinel-2 NDVI plots in 2020 of randomly chosen parcels from each of the representative unique classes identified by the approach in the study region (**a**) Trees/Forest area, (**b**) Urban Area, (**c**) Non-Paddy Crop parcel, (**d**) Parcel with Paddy grown after another crop cycle.

**Figure 16:**
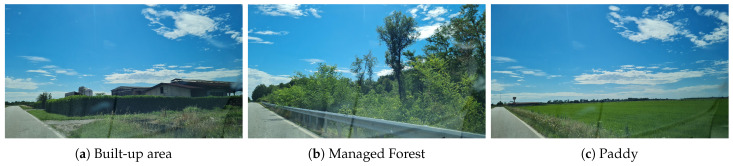
Sample images are drawn from the different output classes of Built-up area (gray), trees/managed forest (dark green) and flooded paddy plots (light green). The images were acquired along Strada Provinciale 20 (https://www.mapillary.com/app/?pKey=1178335279260239, accessed on 16 October 2025) by lahniuscollurio (https://www.mapillary.com/app/user/laniuscollurio, accessed on 16 October 2025), licensed under CC-BY-SA (https://creativecommons.org/licenses/by-sa/4.0/, accessed on 16 October 2025). All the images were acquired in July 2021.

An additional level of verification is by observing the NDVI signatures of each class. We randomly selected a few pixels from each of the following distinct groups: Tree, Urban, Crops, Barren and Flooded Rice, and the NDVI plot is shown in Figure 15. The Figure 15d shows the Copernicus Sentinel-2 multi-spectral satellite-derived vegetation index (NDVI) plot of randomly sampled pixels from the regions classified as Paddy by our approach. The chart shows that the seedling/transplantation from the nursery to the farm plot occurs around the end of April and at the beginning of May, and the vegetative, reproductive, and ripening stages are also clearly visible. As the year of growth and the availability of the data was in 2020, it is difficult to do the ground truth validation, but we verified that the paddy was growing in these plots in 2023. Farmers have traditionally grown paddy in this region for many seasons and will continue to be the main crop in the region. This rice mask is also used as a reference map to validate the results of our classification. In addition to these sources, in the absence of field visit data, we used Street View images to verify selective areas in the regions, which is shown in Figure 16. We also tried the same approach of training a deep learning model with a deep learning model (3D CNN) that uses both spectral and spatial features [58] and then inferred in the Barengo study area, but the model was not transferable and the results were very poor despite the model having a training accuracy of 99.49% and a validation accuracy of 97.24% on the Pines dataset (train:test:val split 60%:20%:20%).

### 3.7. Limitations

There are three current limitations of our proposed approach that warrant discussion:

- Spectral Confusion: The model confuses between spectrally similar crops or same crop with different management practices, and is typical of single temporal snapshot-based analysis. Multi-temporal analysis might resolve this confusion.
- Phenology Sensitivity: The model is sensitivity to the phenological stage and the accuracy is during the maturity stage of the crop, and
- Validation Constraints: The absence of field campaigns for PRISMA dataset means that the methodology depends on proxy validation using multi-sensor cross-validation than actual field campaign data. While we observed that the inter-method agreement was high, having ground-truth field campaigns could also give accuracy statistics.

## 4. Conclusions and Future Work

We propose a novel lightweight framework for Hyperspectral image-based classification by using a combination of pixel-wise supervised and iterative unsupervised learning methods. The key feature is that the learning method, at least, requires estimation of only one parameter and, for a better accuracy, requires a maximum of two parameters and minimal training samples. Across the different benchmark datasets, our method demonstrated 7.22–15% improvements in the overall accuracy compared to pure spectral classifiers. After convergence, the method achieved the highest accuracy in the Salinas dataset (99.417%) and remains comparable to other state-of-the-art models for the Indian Pines dataset (97%). It is best suited for sustainable agricultural applications where ground truth is very sparse and with many unseen classes. The model is faster to train, and the trained model parameters are transferable across the different datasets while maintaining the accuracies. Our lightweight framework offers a robust baseline for domain transfer in label-sparsity situations, though it might not be a replacement for deep networks where data are abundant, diverse, or in well-annotated settings. Transformer-based models such as the MASSFormer [50] work well in closed-set scenarios with >10% training data. We aim to extend this framework to diverse HSI datasets and datasets from a broader set of satellite missions.

## Figures and Tables

**Table 1:** Training data partition comparison between our study and literature.

Dataset	Literature	Our Study	Rationale
Indian Pines	10%	10%	Maintained for comparison
Salinas	4%	1%	Sparse-label stress test
Pavia Univ	6%	1%	Extreme low-resource scenario

**Table 2:** Listing of the optimal parameters required by the joint classifier for each of the benchmark dataset and the final optimal parameter set.

Data	*C*	γ	*r*	$\sigma_{w}$	$\sigma_{s}$	β
Indian Pines	35	8	3	1	1	0.4
Salinas	43	11	9	4	1	0.5
Pavia University	34	7	4	2	2	0.5
Optimal Parameter	Dataset specific	7	4	1.3	1.3	0.4

**Table 3:** Classification accuracy for the different methods on the Indian Pines dataset.

Class	Train	Test	SVM	EPF [48]	IFRF [46]	RMG [49]	Proposed (Single Run)
Alfalfa	20	26	93.48	97.83	100.00	100.00	97.83
Corn-notill	143	1285	77.31	84.45	99.44	95.33	88.17
Corn-mintill	83	747	73.61	91.454	99.76	98.20	91.08
Corn	24	213	54.85	100.00	99.58	97.86	91.14
Grass-pasture	48	435	89.71	97.10	100.00	95.17	92.96
Grass-trees	73	657	96.57	99.73	100.00	98.80	100.00
Grass-pasture-mowed	14	14	96.43	96.43	100.00	100.00	100.00
Hay-windrowed	48	430	94.98	100.00	100.00	100.00	100.00
Oats	10	10	100.00	100.00	100.00	100.00	95.00
Soybean-notill	97	875	79.22	98.35	94.69	98.24	94.03
Soybean-mintill	217	2238	70.06	93.24	98.37	98.58	88.64
Soybean-clean	59	534	65.77	97.98	99.66	98.53	95.28
Wheat	21	184	98.54	99.51	99.51	100.00	100.00
Woods	119	1146	96.52	97.94	99.76	100.00	100.00
Building-grass-trees-drives	39	347	57.25	98.96	99.74	99.74	81.09
Stone-steel-towers	9	84	79.57	100.00	100.00	98.95	97.85
OA (%)			79.30	94.77	99.40	98.23	92.78
AA (%)			82.80	97.06	99.72	98.71	94.57
Kappa			76.50	94.06	99.32	97.99	91.78

**Table 4:** Classification accuracy of the different methods on the Salinas dataset. The results shown for our approach is for a single run and the OA increases to 99.417% for our approach when the single run replaces the spectral classification map.

Class	Train	Test	SVM	EPF [48]	IFRF [46]	RMG [49]	Proposed (Single Run)
broccoli-green-weeds-1	67	1942	99.75	100.00	100.00	100.00	100.00
broccoli-green-weeds-2	67	3659	99.62	100.00	99.95	99.87	100.00
Fallow	67	1909	96.91	100.00	100.00	99.34	100.00
Fallow-rough-plow	69	1325	99.71	99.93	100.00	100.00	98.85
Fallow-smooth	67	2611	96.23	99.29	99.40	98.62	99.63
Stubble	67	3892	99.82	100.00	99.82	99.77	100.00
Celery	68	3511	99.72	99.92	99.83	99.66	99.86
Grapes-untrained	69	11102	63.72	84.74	98.16	98.70	91.78
Soil-vineyard-develop	68	6135	99.69	99.84	99.98	100.00	100.00
Corn-senesced-green-weeds	68	3210	91.31	96.55	99.15	98.84	99.97
Lettuce-romaine-4wk	68	1000	96.72	99.72	100.00	99.81	99.16
Lettuce-romaine-5wk	67	1860	99.69	100.00	100.00	98.44	100.00
Lettuce-romaine-6wk	67	849	98.47	99.13	99.67	99.34	98.80
Lettuce-romaine-7wk	67	1003	94.86	98.60	99.81	99.16	97.01
vineyard-untrained	70	7198	72.76	93.02	98.18	96.82	96.68
vineyard-vertical-trellis	67	1740	98.67	99.23	100.00	100.00	100.00
OA (%)			87.61	95.54	99.22	99.01	97.69
AA (%)			94.23	98.12	99.56	99.27	98.86
Kappa			86.25	95.05	99.13	98.89	97.43

**Table 5:** Classification accuracy of the different methods on the PaviaU dataset. The results are shown for a single run of our approach and increases to 99.44% when the single run replaces the spectral classification map.

Class	Train	Test	SVM	EPF [48]	IFRF [46]	RMG [49]	Our Approach (Single Run)
Asphalt	20	26	88.36	97.19	97.21	99.76	97.48
Meadows	143	1285	93.43	98.46	99.49	99.95	98.6
Gravel	83	747	85.23	91.42	98.52	99.86	94.85
Trees	24	213	96.15	95.50	97.42	97.75	97.29
Painted-metal-sheets	48	435	99.55	100.00	99.63	100.00	100.00
Bare-soil	73	657	92.19	100.00	99.92	100.00	99.54
Bitumen	14	14	94.66	99.92	99.40	100.00	100.00
Self-blocking-bricks	48	430	88.27	99.00	96.66	100.00	96.99
Shadows	10	10	100.00	100.00	99.47	100.00	95.46
OA (%)			92.22	98.06	98.75	99.77	98.14
AA (%)			93.08	97.94	98.64	99.70	97.81
Kappa			89.79	97.44	98.34	99.70	97.55

## Data Availability

For reproducibility, all the data and code will be made available as a github repository at the time of publishing.

## Abbreviations

The following notations and abbreviations are used in this manuscript:

2D	Two-dimensional
3D	Three-dimensional
AA	Average Accuracy
AVIRIS	Airborne Visible/Infrared Imaging Spectrometer
HSI	Hyperspectral Image
CNN	Convolution Neural Network
CV	Cross Validation
DL	Deep Learning
EPF	Edge Preserving Filter
GHG	Green House Gas
GPU	Graphics Processing Unit
HCO	Co-registered Hypercube
ICM	Iterative Conditional Model
IFRF	Image Fusion and Recursive Filtering
kNN	k-Nearest Neighbors
KPCA	Kernel PCA
LSTM	Long Short-Term Memory
MAE	Masked Auto Encoder
ML	Machine Learning
NIR	Near Infrared
OA	Overall Accuracy
PCA	Principal Component Analysis
PRISMA	Hyperspectral Precursor of the Application Mission
RGB	Red-Green-Blue
RBF	Radial Basis Function
RMG	Random Multi-graph
SVM	Support Vector Machine
SWIR	Short-Wave Infrared
UAV	Unmanned Aerial Vehicle
VNIR	Visible and Near-Infrared

## References

[1] Adão T., Hruška J., Pádua L., Bessa J., Peres E., Morais R., Sousa J. (2017). Hyperspectral Imaging: A Review on UAV-Based Sensors, Data Processing and Applications for Agriculture and Forestry. Remote Sens..

[2] Thenkabail P.S., Smith R.B., Pauw E.D. (2000). Hyperspectral Vegetation Indices and Their Relationships with Agricultural Crop Characteristics. Remote Sens. Environ..

[3] Weiss M., Jacob F., Duveiller G. (2020). Remote sensing for agricultural applications: A meta-review. Remote Sens. Environ..

[4] del Cerro J., Cruz Ulloa C., Barrientos A., de León Rivas J. (2021). Unmanned aerial vehicles in agriculture: A survey. Agronomy.

[5] Leslie C.R., Serbina L.O., Miller H.M. (2017). Landsat and Agriculture—Case Studies on the Uses and Benefits of Landsat Imagery in Agricultural Monitoring and Production.

[6] Lu B., Dao P.D., Liu J., He Y., Shang J. (2020). Recent advances of hyperspectral imaging technology and applications in agriculture. Remote Sens..

[7] Arellano P., Tansey K., Balzter H., Boyd D.S. (2015). Detecting the effects of hydrocarbon pollution in the Amazon forest using hyperspectral satellite images. Environ. Pollut..

[8] Apan A., Held A., Phinn S., Markley J. (2004). Detecting sugarcane ‘orange rust’ disease using EO-1 Hyperion hyperspectral imagery. Int. J. Remote Sens..

[9] Berger K., Verrelst J., Féret J.B., Wang Z., Wocher M., Strathmann M., Danner M., Mauser W., Hank T. (2020). Crop nitrogen monitoring: Recent progress and principal developments in the context of imaging spectroscopy missions. Remote Sens. Environ..

[10] Persello C., Wegner J.D., Hänsch R., Tuia D., Ghamisi P., Koeva M., Camps-Valls G. (2022). Deep learning and earth observation to support the sustainable development goals: Current approaches, open challenges, and future opportunities. IEEE Geosci. Remote Sens. Mag..

[11] Khan M.J., Khan H.S., Yousaf A., Khurshid K., Abbas A. (2018). Modern Trends in Hyperspectral Image Analysis: A Review. IEEE Access.

[12] Signoroni A., Savardi M., Baronio A., Benini S. (2019). Deep Learning Meets Hyperspectral Image Analysis: A Multidisciplinary Review. J. Imaging.

[13] Audebert N., Le Saux B., Lefevre S. (2019). Deep Learning for Classification of Hyperspectral Data: A Comparative Review. IEEE Geosci. Remote Sens. Mag..

[14] Chen Y., Zhao X., Jia X. (2015). Spectral–Spatial Classification of Hyperspectral Data Based on Deep Belief Network. IEEE J. Sel. Top. Appl. Earth Obs. Remote Sens..

[15] Zhang L., Zhang L., Du B. (2016). Deep Learning for Remote Sensing Data: A Technical Tutorial on the State of the Art. IEEE Geosci. Remote Sens. Mag..

[16] Zhong Z., Li J., Luo Z., Chapman M. (2017). Spectral-Spatial Residual Network for Hyperspectral Image Classification: A 3-D Deep Learning Framework. IEEE Trans. Geosci. Remote Sens..

[17] Li Y., Zhang H., Shen Q. (2017). Spectral-Spatial Classification of Hyperspectral Imagery with 3D Convolutional Neural Network. Remote Sens..

[18] Gao Q., Lim S., Jia X. (2018). Hyperspectral Image Classification Using Convolutional Neural Networks and Multiple Feature Learning. Remote Sens..

[19] Kemker R., Salvaggio C., Kanan C. (2018). Algorithms for semantic segmentation of multispectral remote sensing imagery using deep learning. ISPRS J. Photogramm. Remote Sens..

[20] Guidici D., Clark M. (2017). One-Dimensional Convolutional Neural Network Land-Cover Classification of Multi-Seasonal Hyperspectral Imagery in the San Francisco Bay Area, California. Remote Sens..

[21] Bach S., Binder A., Montavon G., Klauschen F., Müller K.R., Samek W. (2015). On Pixel-Wise Explanations for Non-Linear Classifier Decisions by Layer-Wise Relevance Propagation. PLoS ONE.

[22] Montavon G., Lapuschkin S., Binder A., Samek W., Müller K.R. (2017). Explaining nonlinear classification decisions with deep Taylor decomposition. Pattern Recognit..

[23] Liu Y., Tang Y., Zhang L., Liu L., Song M., Gong K., Peng Y., Hou J., Jiang T. (2020). Hyperspectral open set classification with unknown classes rejection towards deep networks. Int. J. Remote Sens..

[24] Yue J., Fang L., He M. (2022). Spectral-spatial latent reconstruction for open-set hyperspectral image classification. IEEE Trans. Image Process..

[25] Aziz A., Bahrudeen A., Rahim A. (2024). Spectral Fidelity and Spatial Enhancement: An Assessment and Cascading of Pan-Sharpening Techniques for Satellite Imagery. arXiv.

[26] Alparone L., Arienzo A., Garzelli A. (2024). Spatial Resolution Enhancement of Satellite Hyperspectral Data via Nested Hypersharpening With Sentinel-2 Multispectral Data. IEEE J. Sel. Top. Appl. Earth Obs. Remote Sens..

[27] Zhu L., Wu J., Biao W., Liao Y., Gu D. (2023). SpectralMAE: Spectral Masked Autoencoder for Hyperspectral Remote Sensing Image Reconstruction. Sensors.

[28] Meng Z., Yan Q., Zhao F., Liang M. Hyperspectral Image Classification with Dynamic Spatial-Spectral Attention Network. Proceedings of the 2023 13th Workshop on Hyperspectral Imaging and Signal Processing: Evolution in Remote Sensing (WHISPERS).

[29] Kang J., Zhang Y., Liu X., Cheng Z. (2024). Hyperspectral Image Classification Using Spectral–Spatial Double-Branch Attention Mechanism. Remote Sens..

[30] Ahmad M., Distifano S., Khan A.M., Mazzara M., Li C., Yao J., Li H., Aryal J., Vivone G., Hong D. (2024). A Comprehensive Survey for Hyperspectral Image Classification: The Evolution from Conventional to Transformers. arXiv.

[31] Datta A., Ghosh S., Ghosh A., Naik G.R. (2018). PCA, Kernel PCA and Dimensionality Reduction in Hyperspectral Images. Advances in Principal Component Analysis: Research and Development.

[32] Wold S., Esbensen K., Geladi P. (1987). Principal component analysis. Chemom. Intell. Lab. Syst..

[33] Licciardi G., Marpu P.R., Chanussot J., Benediktsson J.A. (2012). Linear Versus Nonlinear PCA for the Classification of Hyperspectral Data Based on the Extended Morphological Profiles. IEEE Geosci. Remote Sens. Lett..

[34] Roy S., Krishna G., Dubey S.R., Chaudhuri B. (2019). HybridSN: Exploring 3-D-2-D CNN Feature Hierarchy for Hyperspectral Image Classification. IEEE Geosci. Remote Sens. Lett..

[35] Hermes L., Frieauff D., Puzicha J., Buhmann J.M. Support vector machines for land usage classification in Landsat TM imagery. Proceedings of the IEEE 1999 International Geoscience and Remote Sensing Symposium, IGARSS’99 (Cat. No.99CH36293).

[36] Melgani F., Bruzzone L. (2004). Classification of hyperspectral remote sensing images with support vector machines. IEEE Trans. Geosci. Remote Sens..

[37] Wen Z., Shi J., Li Q., He B., Chen J. (2018). ThunderSVM: A Fast SVM Library on GPUs and CPUs. J. Mach. Learn. Res..

[38] Shotton J., Winn J.M., Rother C., Criminisi A. (2009). TextonBoost for Image Understanding: Multi-Class Object Recognition and Segmentation by Jointly Modeling Texture, Layout, and Context. Int. J. Comput. Vis..

[39] Krähenbühl P., Koltun V. (2012). Efficient Inference in Fully Connected CRFs with Gaussian Edge Potentials. arXiv.

[40] Zhang W., Li M. (2014). MRF and CRF Based Image Denoising and Segmentation. Proceedings of the 2014 5th International Conference on Digital Home.

[41] Staubitz T., Klement H., Teusner R., Renz J., Meinel C. CodeOcean—A versatile platform for practical programming excercises in online environments. Proceedings of the 2016 IEEE Global Engineering Education Conference (EDUCON).

[42] Wang S. (2020). Hyperspectral Dataset. https://ieee-dataport.org/documents/hyperspectral-dataset.

[43] Baumgardner M.F., Biehl L.L., Landgrebe D.A. (2015). 220 Band AVIRIS Hyperspectral Image Data Set: 12 June 1992 Indian Pine Test Site 3. https://purr.purdue.edu/publications/1947/1.

[44] Hsu C.W., Chang C.C., Lin C.J. (2003). A Practical Guide to Support Vector Classification.

[45] Ben-Hur A., Weston J. (2010). A user’s guide to support vector machines. Methods Mol. Biol..

[46] Kang X., Li S., Benediktsson J.A. (2014). Feature Extraction of Hyperspectral Images With Image Fusion and Recursive Filtering. IEEE Trans. Geosci. Remote Sens..

[47] Chapelle O., Vapnik V., Bousquet O., Mukherjee S. (2002). Choosing Multiple Parameters for Support Vector Machines. Mach. Learn..

[48] Kang X., Li S., Benediktsson J.A. (2014). Spectral–Spatial Hyperspectral Image Classification with Edge-Preserving Filtering. IEEE Trans. Geosci. Remote Sens..

[49] Gao F., Wang Q., Dong J., Xu Q. (2018). Spectral and Spatial Classification of Hyperspectral Images Based on Random Multi-Graphs. Remote Sens..

[50] Sun L., Zhang H., Zheng Y., Wu Z., Ye Z., Zhao H. (2024). MASSFormer: Memory-Augmented Spectral-Spatial Transformer for Hyperspectral Image Classification. IEEE Trans. Geosci. Remote Sens..

[51] Hao P., Di L., Zhang C., Guo L. (2020). Transfer Learning for Crop classification with Cropland Data Layer data (CDL) as training samples. Sci. Total Environ..

[52] Wang Y., Feng L., Zhang Z., Tian F. (2023). An unsupervised domain adaptation deep learning method for spatial and temporal transferable crop type mapping using Sentinel-2 imagery. ISPRS J. Photogramm. Remote Sens..

[53] Zanaga D., Van De Kerchove R., De Keersmaecker W., Souverijns N., Brockmann C., Quast R., Wevers J., Grosu A., Paccini A., Vergnaud S. (2021). ESA WorldCover 10 m 2020 v100. https://zenodo.org/records/5571936.

[54] Candela L., Formaro R., Guarini R., Loizzo R., Longo F., Varacalli G. The PRISMA mission. Proceedings of the 2016 IEEE International Geoscience and Remote Sensing Symposium (IGARSS).

[55] Qian H., Zhu X., Huang S., Linquist B., Kuzyakov Y., Wassmann R., Minamikawa K., Martinez-Eixarch M., Yan X., Zhou F. (2023). Greenhouse gas emissions and mitigation in rice agriculture. Nat. Rev. Earth Environ..

[56] Büttner G., Manakos I., Braun M. (2014). CORINE Land Cover and Land Cover Change Products. Land Use and Land Cover Mapping in Europe: Practices & Trends.

[57] Singha M., Dong J., Zhang G., Xiao X. (2019). High resolution paddy rice maps in cloud-prone Bangladesh and Northeast India using Sentinel-1 data. Sci. Data.

[58] Ahmad M., Khan A.M., Mazzara M., Distefano S., Ali M., Sarfraz M.S. (2022). A Fast and Compact 3-D CNN for Hyperspectral Image Classification. IEEE Geosci. Remote Sens. Lett..

